# Food waste composition, hotspots and sustainability impacts in an airline catering kitchen

**DOI:** 10.1177/0734242X261455573

**Published:** 2026-06-15

**Authors:** Niina Sundin, Tahir Babatunde, Viachaslau Filimonau, Mattias Eriksson, Christopher Malefors

**Affiliations:** 1 Department of Energy and Technology, Swedish University of Agricultural Sciences, Uppsala, Sweden; 2 Business School, University of Surrey, Stag Hill, University Campus, Guildford, Sweden

**Keywords:** waste audit, edible waste, waste prevention, carbon footprint, aviation food service, airline catering, nutrient loss, operational efficiency

## Abstract

Aviation generates substantial food waste worldwide, yet the focus has mainly been on post-flight waste, leaving upstream production processes in airline catering understudied. This mixed-methods single case study investigated food waste composition, hotspots and drivers in a Swedish airline catering kitchen, and assessed associated environmental (carbon), economic (procurement cost) and social (nutritional) impacts. Over 21 weekdays, waste audits recorded 1.39 tonnes of food waste, largely edible (89%), corresponding to 3.1 tonnes of carbon dioxide equivalents, €9250 in procurement costs, and 93 kg of protein and 29 kg of dietary fibre losses. Hot kitchen operations, trolley waste and crew meals were the main hotspots, accounting for most edible waste and associated impacts. A divergence between volume-driven and intensity-driven hotspots was found, with vegetables and staple foods driving economic losses and fibre waste, and animal-based foods dominating climate impacts and protein losses. Notably, staple foods emerged as a balanced hotspot across several impact dimensions. Semi-structured staff interviews (*n* = 8) identified overly strict food safety routines, late airline and crew changes, forecasting mismatches, and coordination gaps as key structural drivers of waste, leading to recurrent overproduction and limited flexibility in meal handling. By shifting attention from downstream to upstream, the study shows that targeting kitchen-stage processes offers a viable route to reducing avoidable waste by addressing systemic inefficiencies and rigid safety buffers. Integrating contractual cut-off times for order changes into procurement and governance measures that encourage shared responsibility for waste prevention offers a pathway to improving resource efficiency while maintaining food safety and service standards.

## Introduction

Each year, the global airline industry serves meals to millions of passengers while balancing strict food safety regulations, uncertain passenger numbers, and rigid service schedules (Rajaratnam and Sunmola, 2021b). These same factors that enable safe and reliable service also contribute to the generation of food waste, much of which is edible and resource-intensive to produce (Thamagasorn and Pharino, 2019). Food waste is a critical global sustainability issue, linked to greenhouse gas emissions, resource depletion, economic loss, and food insecurity (FAO, 2013; UNEP, 2024). Addressing food waste in aviation is therefore not only an operational issue but also a major sustainability concern (Heydari et al., 2025). While aviation is widely recognised as a carbon-intensive sector, with fuel combustion dominating its greenhouse gas emissions profile, decarbonising aviation will require long development timelines and substantial investment in aircraft efficiency and fuel substitution (IATA, 2024; IPCC, 2022). By contrast, food waste reduction in airline catering operations offers an immediately implementable and relatively low-cost opportunity to shrink the sector’s broader environmental footprint (You et al., 2024). Food waste prevention thus represents a practical lever for the aviation industry to advance towards its sustainability and decarbonisation targets.

Despite its relevance, food waste prevention in airline catering has received comparatively limited research attention. Food waste has been relatively well studied in restaurants, households, and public meal settings, such as school canteens and hospitals (Eriksson et al., 2017a; Sjölund et al., 2025; Sundin et al., 2024b). In aviation, early work, such as El-Mobaidh et al. (2006), classified waste generated during in-flight catering on Egypt Air flights, revealing food residues as a major component of cabin waste. Subsequent studies have largely concentrated on post-flight cabin waste management, including uneaten meals, packaging, and service items collected after landing (Blanca-Alcubilla et al., 2018). A related stream of research has examined food waste management in airport settings (Baxter et al., 2018; Lam et al., 2018). Far less is known about food waste generated before departure, that is, within the production kitchens where meals are planned, cooked, and portioned for specific flights, as these studies remain limited in analytical scope, focusing primarily on quantifying food waste and identifying proximate causes (Thamagasorn and Pharino, 2019).

To understand and address the challenge of food waste effectively, it is essential to go beyond quantification. Due to the focus on quantitative assessments, previous studies have left the organisational and behavioural factors behind the waste poorly understood. Capturing such operational dynamics requires in-depth, qualitative insights from staff directly involved in food production and waste handling. Moreover, a detailed composition analysis can reveal dominant food fractions and operational patterns, enabling targeted interventions rather than generic reduction targets (Papargyropoulou et al., 2014). Most existing waste composition studies have, however, focused on post-flight cabin waste rather than on waste generated in production kitchens, where prevention opportunities are shaped by catering operations rather than passenger behaviour outside the caterer’s control (Blanca-Alcubilla et al., 2018; El-Mobaidh et al., 2006). Further, a hotspot analysis complements the aforementioned by locating the sections, waste categories, or meal types responsible for the largest losses, allowing prioritisation of preventive actions where impact is greatest (Eriksson et al., 2017a). Together, these approaches can be enriched by a sustainability assessment across environmental, economic, and social dimensions to evaluate the broader consequences of edible food waste and the potential co-benefits and trade-offs of its prevention (Goossens et al., 2019; Spiker et al., 2017). However, despite the increasing attention to the sustainability impacts of food waste, most existing assessments retain their focus on environmental indicators, typically greenhouse gas emissions, while occasionally including economic impacts, but seldom addressing social dimensions, such as nutrient losses or labour effects (Goossens et al., 2019; Lehn and Schmidt, 2023; Ranundeniya et al., 2025). Recent work has therefore emphasised the need for more integrated sustainability assessments that jointly consider environmental, economic and social outcomes (Nabavi-Pelesaraei and Hamidinasab, 2025).

Responding to these knowledge gaps, this study aimed to investigate food waste generation in the production kitchen of a Swedish branch of a global airline catering company. It combined quantitative waste audits with in-depth staff interviews to analyse waste composition, hotspots, and drivers of edible food waste. Beyond quantification, the study assessed the sustainability implications of the edible fraction of waste through a triple-bottom-line lens: environmental impacts (carbon footprint), economic impacts (procurement costs), and social value (nutrient losses). The interpretation of the social dimension follows Goossens et al. (2019) and Wang et al. (2023), and aligns with FAO’s sustainability framework, which situates access to safe and nutritious food within the social well-being pillar (Scialabba, 2014; Swedish FAO Committee, 2021). Including protein and dietary fibre losses thus operationalises the social dimension of sustainability, consistent with emerging approaches that integrate nutrition and health into hospitality sustainability assessments (Salehi et al., 2025). This integrated approach aims to provide a richer understanding of where and how prevention efforts can deliver the greatest sustainability gains, informing both operational decision-making and sector-wide strategies for resource efficiency.

The present study was guided by the following questions:

1. What is the composition and mass of food waste generated in an airline catering production kitchen across its three main subsections?
2. Where are the main hotspots of waste generation across kitchen subsections, meal types, and food categories?
3. What operational and organisational factors drive food waste in this context, and what are the associated sustainability implications?

This study contributes to the literature in three ways. First, it shifts the analytical focus from post-flight waste to food waste generated upstream in airline catering production, addressing a stage that has received limited empirical attention. Second, it integrates waste quantification with environmental, economic, and nutritional assessments, demonstrating how different food categories dominate different sustainability dimensions and revealing trade-offs that are obscured in single-indicator analyses. Third, by combining waste audits with staff interviews, the study links operational and organisational drivers to cross-dimensional sustainability impacts, identifying prevention leverage points that are structurally embedded in airline catering systems rather than specific to an individual case.

## Materials and methods

### Study design and approach

This study used a concurrent mixed-methods case study design, combining quantitative and qualitative data (Collins et al., 2007). Quantitative and qualitative data were collected during the same period, in March 2025, analysed separately, and then integrated to provide a comprehensive understanding of both the scale and underlying causes of waste. The quantitative component consisted of a waste audit to measure daily volumes and composition of food waste by kitchen section, meal type, food category, and edibility. The qualitative part consisted of semi-structured staff interviews to explore operational practices, perceived drivers of waste, and existing reduction measures. A case study design was considered appropriate given the exploratory nature of the research and the need to examine food waste generation within its real-world operational context. The selected facility provided access to detailed production, waste, and staff-perspective data across multiple kitchen functions, enabling in-depth analysis of upstream food waste processes that remain underexplored in airline catering.

Integration of findings occurred at the interpretation stage through methodological triangulation, where qualitative insights were used to explain quantitative waste patterns, enhancing the robustness of interpretation (Filimonau et al., 2023; Papargyropoulou et al., 2016). The study was conducted over 21 consecutive weekdays (Monday–Friday), excluding weekends in March 2025, representing the low travel season. Although seasonal variation cannot be excluded, the period was considered indicative of routine weekday operations rather than atypical peak-season conditions.

### Site description

The case company was selected through convenience sampling. The facility is part of a large international catering group that provides in-flight meals to multiple airlines operating domestic, European, and intercontinental routes. The unit employs around 125 staff during regular operations, with additional temporary workers hired during peak travel periods (May–July).

The centralised production kitchen comprises three main subsections:

1. Hot kitchen – prepares hot meals using a cook–chill process.
2. Cold kitchen (including plating room) – assembles cold dishes, desserts, and final plating.
3. Slicing room – decants and prepares toppings, condiments, and other meal components.

The facility produces approximately 12,000–15,000 meals per week, including crew meals, business-class menus, and services for PLUS-class passengers, as well as occasional chartered flights. During the study period, the facility served approximately five to six short-haul flights and one long-haul flight per day, with additional charter flights (one to five flights) operating between Friday and Sunday. On average, around 2000 in-house meals (including hot meals, cold meals, salads, desserts, and fruit items) were prepared daily, depending on route and service class. The dataset covered both short- and long-haul flights; however, business-class waste originated exclusively from long-haul flights. Economy-class meals for long-haul flights were partially prepared externally, with the main meal components typically outsourced, and were therefore excluded from the analysis, while accompanying components (e.g., salads, desserts, and breakfast items) prepared in-house were included. The proportion of externally prepared meal components could not be reliably quantified based on the available data and is therefore acknowledged as a limitation of the study.

A substantial but not quantified share of ingredients was procured from local suppliers, in line with the company’s stated goal of achieving full local sourcing. While the study is based on a single facility, it reflects key operational characteristics described for airline catering systems, including centralised and standardised multi-stage workflows, coordination across multiple actors, the use of cook–chill production, and strict food safety requirements under time-sensitive conditions (European Commission, 2004; Rajaratnam and Sunmola, 2021a, 2021b).

### Definitions and waste categories

Food waste was defined according to the EU common methodology for food waste measurement as any food and inedible parts removed from the food supply chain for recovery or disposal, including composting, anaerobic digestion, or energy recovery (European Commission, 2020). Waste was classified as edible (intended for human consumption) or inedible (e.g., bones, peels) at the point of discard. Edible waste included all food originally intended for consumption, regardless of condition.

Three operational waste categories were used:

1. Preparation waste: spoilage and food discarded during ingredient preparation or cooking.
2. Portioning waste: surplus generated during meal assembly and plating.
3. Trolley waste: cooked food stored in trays in refrigerated trolleys that passed their use-by date before service.

### Waste audit procedure

Waste audits were conducted daily across the three kitchen subsections by the same researcher following a consistent protocol for sorting and categorisation. This approach ensured the uniform application of classification criteria across the entire audit period. However, the use of a single assessor precludes formal assessment of inter-rater reliability, which may introduce some degree of subjectivity in waste categorisation. Prior to the task, the researcher had received training in food waste weighing. All waste was weighed using a calibrated electronic scale (precision ±1 g), supporting consistency in measurement and recording. However, no formal validation procedures (e.g., duplicate measurements or inter-rater checks) were conducted, which may introduce some uncertainty in the data.

Waste was intercepted at three main points: (1) directly from trays of expired or unused cooked food, (2) from preparation stations at the point of discard, and (3) from mis-sorted waste bins, where food was retrieved, sorted, and reclassified. In some cases, waste could not be reliably assigned to a specific operational category (preparation, portioning, or trolley waste). This occurred primarily when food waste had already been mixed prior to collection, making it impossible to determine its original point of generation. Such instances were therefore classified as an “unknown” waste category.

The researcher was present during daily operations and monitored waste flows across kitchen sections, including checks of additional waste bins, to capture all major waste streams. Nevertheless, as the audit relied on manual interception and sorting, minor quantities of waste may have bypassed measurement. Weights were recorded in grams alongside the food item, meal type, kitchen section, waste category, and edibility status. Food items were classified into 12 internally consistent food categories (bread, staple foods, vegetables, fruits, dairy, poultry, fish and seafood, meat, meat alternatives, mixed foods, sauces, and desserts). These categories were designed to reflect the major food groups represented in airline catering menus while maintaining conceptual alignment with the EU’s common food group structure for food waste reporting (European Commission, 2020). Food status was recorded as fresh, in-house prepared, pre-prepared, or ready-to-eat.

### Interviews

Semi-structured interviews were conducted with eight staff members representing management, production, and supply functions. Participants were purposively selected to capture a range of roles and perspectives across the production workflow, ensuring coverage of the kitchen’s main operational processes. Additional selection criteria included participants’ ability to communicate in English, as the interviews were conducted in English, and their willingness to participate. This may have excluded perspectives from non-English-speaking staff, potentially limiting the completeness of the qualitative findings, particularly among operational roles. Although the number of participants was modest, thematic saturation was achieved within the interviewed sample, that is, no new themes emerged in the final interviews, falling within the typical range for saturation in qualitative research (Fusch and Ness, 2015). Thematic saturation was operationally defined as the point at which no new codes or themes emerged from the interview data. Coding was conducted following data collection, and saturation was assessed during the coding process, with the final interviews not yielding additional thematic insights.

The interview guide (Supplemental Material S1) was designed to elicit detailed insights into the perceived causes and departmental dynamics contributing to food waste, enabling participants to articulate both the “what” and the “why” of waste generation within their respective roles. Example questions included: “What do you think makes it difficult to reduce food waste in your department?,” “Have any actions been taken to reduce food waste, and did they help?.” The interviews were conducted using a semi-structured approach, allowing flexibility to follow up on relevant topics raised by participants. Additional probing questions were asked where appropriate, related to issues such as food safety regulations, organisational policies, and operational constraints. Participation was voluntary, with informed consent obtained prior to audio recording. Interviews lasted 30–75 minutes and were conducted in private meeting rooms to minimise disruption and encourage candid responses. All interviews were transcribed verbatim, and transcripts were anonymised to ensure confidentiality. Ethical approval was not required under the Swedish Ethical Review Act (2003:460), as the study did not involve sensitive personal data.

### Data analysis

Quantitative data were analysed descriptively to calculate daily waste generation, composition by food item and category, division by meal type, distribution by kitchen section, and waste categorisation, as well as the proportion of edible versus inedible fractions to identify edible waste hotspots. Results were expressed as absolute weights and percentages of total waste. For daily edible food waste, the average, standard deviation, and range were calculated to characterise day-to-day variability.

Qualitative data were analysed thematically following the six-phase framework proposed by Braun and Clarke (2006). Coding was conducted by a single researcher using a manual approach without specialised qualitative analysis software. No formal assessment of inter-coder reliability was conducted. Codes were developed both inductively and deductively: while the initial framework was informed by established models of food waste generation in the foodservice sector (e.g., Papargyropoulou et al., 2016), new codes emerged directly from the transcripts and were subsequently integrated into the analysis. Through iterative review and comparison, codes were grouped into overarching themes that captured key organisational, operational, and systemic factors underlying food waste generation. Findings from the qualitative analysis were subsequently triangulated with quantitative waste audit data to identify converging hotspots and explanatory patterns.

To protect confidentiality, the anonymised transcripts were assigned unique identifiers (JD I–VIII), corresponding to individual participants. These identifiers are used in parentheses following quotations to indicate the range of perspectives represented. All quotations presented in the findings are verbatim extracts from anonymised staff interviews, edited only for minor linguistic clarity.

### Sustainability assessment

The sustainability implications of edible food waste were evaluated using a framework encompassing environmental, economic, and social dimensions, consistent with the FAO SAFA Guidelines (Scialabba, 2014) and recent applications in food waste-prevention assessments (Goossens et al., 2019; Wang et al., 2023). The framework applies the triple-bottom-line approach, in which sustainability in food systems is assessed through indicators of resource efficiency, economic viability, and human well-being.

The environmental dimension was represented by the carbon footprint of wasted food, the economic by procurement costs, and the social by the nutritional value of the discarded edible fraction, quantified as protein and dietary fibre losses. These metrics capture the societal value of food waste as potential nourishment, translating food waste into forgone nutrients essential for health and food security. Protein, as an essential nutrient, indicates nutritional adequacy and social equity (Spiker et al., 2017). Fibre represents dietary quality of public-health relevance, as it remains under-consumed in high-income populations (Nordic Council of Ministers, 2023).

As hospitality and catering services link food preparation and consumption, their sustainability performance should address not only resource efficiency but also healthier, more sustainable diets (Salehi et al., 2025). Integrating nutritional indicators, therefore, extends traditional environmental–economic assessments and strengthens the social pillar of sustainability, echoing recent calls to include health aspects in hospitality evaluations.

#### Environmental impact assessment

The greenhouse gas emissions associated with the edible fraction of the food waste were estimated by multiplying the mass of each discarded food item by its corresponding emission factor (kilogram CO_2_-eq per kilogram product). Emission factors were sourced from the SAFAD database, which provides consumption-weighted averages for foods on the Swedish market (Röös et al., 2025; Swedish University of Agricultural Sciences, 2024; Supplemental Table S2). These values account for the origin of raw commodities (domestic vs imported shares), production-related losses, as well as waste occurring at the retailer and consumer stages. Thus, the footprints represent an aggregated perspective of average Swedish food consumption, rather than a single production site or country of origin. Moreover, a cradle-to-retail boundary was applied, that is, upstream production impacts were included, while transport to the catering facility and end-of-life management of the waste were excluded. Categories were matched to representative food items, prioritising cooked values for prepared food and raw values for fresh waste. Where necessary, category-level averages were used.

#### Economic impact assessment

Economic losses from edible food waste were estimated by multiplying the discarded mass of each food item by its corresponding unit price (SEK per kilogram product). Procurement price data were primarily provided by the catering company but are not disclosed due to commercial confidentiality constraints, which limit independent verification of the economic estimates. For a subset of items where procurement records were incomplete or missing, category-level averages were used. All prices were expressed in Swedish kronor (SEK) and then converted to approximate EUR prices and reflect the cost to the catering company at 2025 values, not retail value.

#### Social impact assessment

Nutrient content per 100 g for each food category was obtained from the Swedish Food Agency’s (2025) food composition database (Livsmedelsdatabasen; Supplemental Table S3). Each category was matched to a representative food item, prioritising cooked values for prepared food and raw values for fresh waste. The nutrient losses of protein and dietary fibre were then calculated by multiplying nutrient content by the corresponding mass of discarded edible food.

## Results

### Overall waste generation

Over the 21-day study period, a total of 1.39 tonnes of solid waste was recorded from the production kitchen, equivalent to 10% of total food produced (14.4 tonnes; Table 1). Of this, almost 90% was classified as edible food waste. This edible fraction accounted for roughly 3.1 tonnes CO_2_-eq in climate impact and about 102 kSEK (9250 EUR) in procurement costs. Daily edible waste averaged 59.3 ± 18.3 kg/day, with a range of 29.7–103.6 kg/day. This corresponded to about 150 kg CO_2_-eq and 440 EUR per day. In nutrient terms, the discarded edible food contained around 93 kg of protein and 29 kg of dietary fibre. To put the daily nutrient losses into perspective, the protein losses corresponded to 190–260 adult meals, and the dietary fibre losses to 125–175 adult meals per day (Nordic Council of Ministers, 2023).

**Table 1.** The amount of food waste and its impacts across the study period, as well as daily averages. The sustainability impacts are also expressed per kilogram of edible food waste.

Indicator	Study period	Daily average	Per kilogram edible waste
Total food produced (kg)	14,409	686.1	n/a
Total food waste (kg)	1392	66.3	n/a
Total food waste (% of production)	9.7	9.7	n/a
Edible waste (kg)	1245	59.3	n/a
Inedible waste (kg)	147	7.0	n/a
Carbon footprint (kg CO_2_-eq)	3070	146	2.5
Economic loss (SEK (EUR))	102,070 (9250)	4860 (440)	82.0 (7.4)
Protein loss (kg)	92.9	4.4	0.07
Dietary fibre loss (kg)	29.1	1.4	0.02

Of the inedible fraction, 77% (113 kg) originated from the slicing room, and the remaining 23% (34 kg) from the hot kitchen. However, since the majority of the waste originated from food intended for human consumption and can be considered avoidable, the edible fraction was kept as the main focus of further analysis.

### Waste hotspots by production section

The three production areas, hot kitchen, cold kitchen, and slicing room, showed distinct edible food waste profiles. The hot kitchen generated the largest share, with 788 kg (63% of the total edible waste), followed by the slicing room with 414 kg (33%), while the cold kitchen contributed only marginally (Table 2). Daily variability was highest in the hot kitchen (37.5 ± 17.4 kg/day), followed by the slicing room (19.7 ± 9.3 kg/day) and the cold kitchen (2.5 ± 1.8 kg/day). When sustainability impacts were considered, the hot kitchen likewise dominated across all impact dimensions, accounting for roughly two-thirds of the total carbon footprint and most of the associated economic and nutrient losses (Table 2). The slicing room produced about one-third of the impacts, whereas the cold kitchen contributed approximately 6% or less. These patterns identify the hot kitchen as the principal hotspot for targeted waste-prevention measures.

**Table 2.** Edible food waste and associated sustainability impacts across production sections, waste categories, and meal types in the airline catering kitchen. Values show absolute amounts and relative shares (%) of total edible food waste across five impact dimensions: waste mass, carbon footprint, economic loss, and nutrient losses (protein and dietary fibre). Shaded cells indicate recurrent hotspots across impact dimensions: hot kitchen, trolley waste, and crew meals, which accounted for most edible waste and sustainability impacts. Other = minor meal categories, including child, PLUS-class and special meals. A heatmap shows sustainability impacts of food categories, sorted by total waste, with a focus on edible waste and three sustainability dimensions.

Impact category	Edible waste kilogram (%)	Carbon footprint kilogram CO_2_-eq (%)	Economic loss^a^ EUR (%)	Protein loss kilogram (%)	Fibre loss kilogram (%)
Production section					
Hot kitchen	788 (63)	2030 (66)	5440 (59)	65 (70)	19 (63)
Slicing room	414 (33)	930 (30)	3260 (35)	25 (27)	10 (33)
Cold kitchen	43 (3)	110 (4)	550 (6)	3 (3)	1 (3)
Waste category					
Trolley	697 (56)	1920 (63)	5360 (58)	57 (62)	16 (55)
Preparation	341 (27)	695 (23)	2545 (27)	22 (24)	8 (28)
Portioning	68 (5)	157 (5)	455 (5)	7 (7)	1 (3)
Unknown^b^	138 (11)	300 (10)	910 (10)	7 (7)	4 (14)
Meal type					
Crew	660 (53)	1385 (45)	5035 (54)	54 (59)	17 (61)
Business	300 (24)	1213 (39)	2200 (23)	24 (26)	6 (21)
Other	282 (23)	475 (15)	2130 (23)	13 (14)	5 (18)

a Procurement prices are confidential; category-level economic estimates cannot be independently verified.

b The “unknown” category reflects waste for which the point of origin within the production process could not be determined due to prior mixing of waste streams.

### Waste hotspots by waste category

When analysed by waste category, trolley waste was the dominant source across all kitchen sections, accounting for about 700 kg (56%) of total edible waste (Table 2). It was particularly prominent in the hot kitchen, where large volumes of prepared meals (484 kg, 39% of total waste) were stored in trolleys awaiting service. Preparation waste was also substantial, roughly one-quarter of total losses, reflecting the intensive handling of ingredients in these areas, whereas portioning waste remained minor (Table 2). Across sustainability dimensions, trolley waste was responsible for over 60% of the total climate impact and a similar magnitude of economic and nutrient losses (Table 2). Preparation waste contributed about one-quarter of total impacts, while portioning waste was minor across all categories. Together, these patterns confirm trolley waste as the principal multi-dimensional hotspot, with preparation waste as a secondary priority for prevention.

### Waste hotspots by meal type

Edible food waste distribution also varied by meal type. Crew meals represented the largest share (660 kg, 53% of edible waste), followed by business-class meals (300 kg, 24%; Table 2). Other categories, such as child, PLUS, and special meals, contributed comparatively little. In terms of sustainability impacts, crew and business meals together generated roughly 80% of the total climate impact and economic and nutrient losses, indicating that waste from these two categories dominated overall performance. Although crew-meal waste occurred in greater quantities, driving the largest sustainability impacts, the total carbon footprints of crew and business meals were quite similar, owing to the higher carbon intensity of business-class meals (roughly 4 kg vs 2 kg CO_2_-eq per kilogram edible waste). This difference reflects the greater resource content of business-class menus. These patterns confirm that waste associated with crew and business meals represents the key hotspot across meal types, driven by high waste volumes in the former and high carbon impact intensity in the latter.

### Food category breakdown

**Figure 1.**
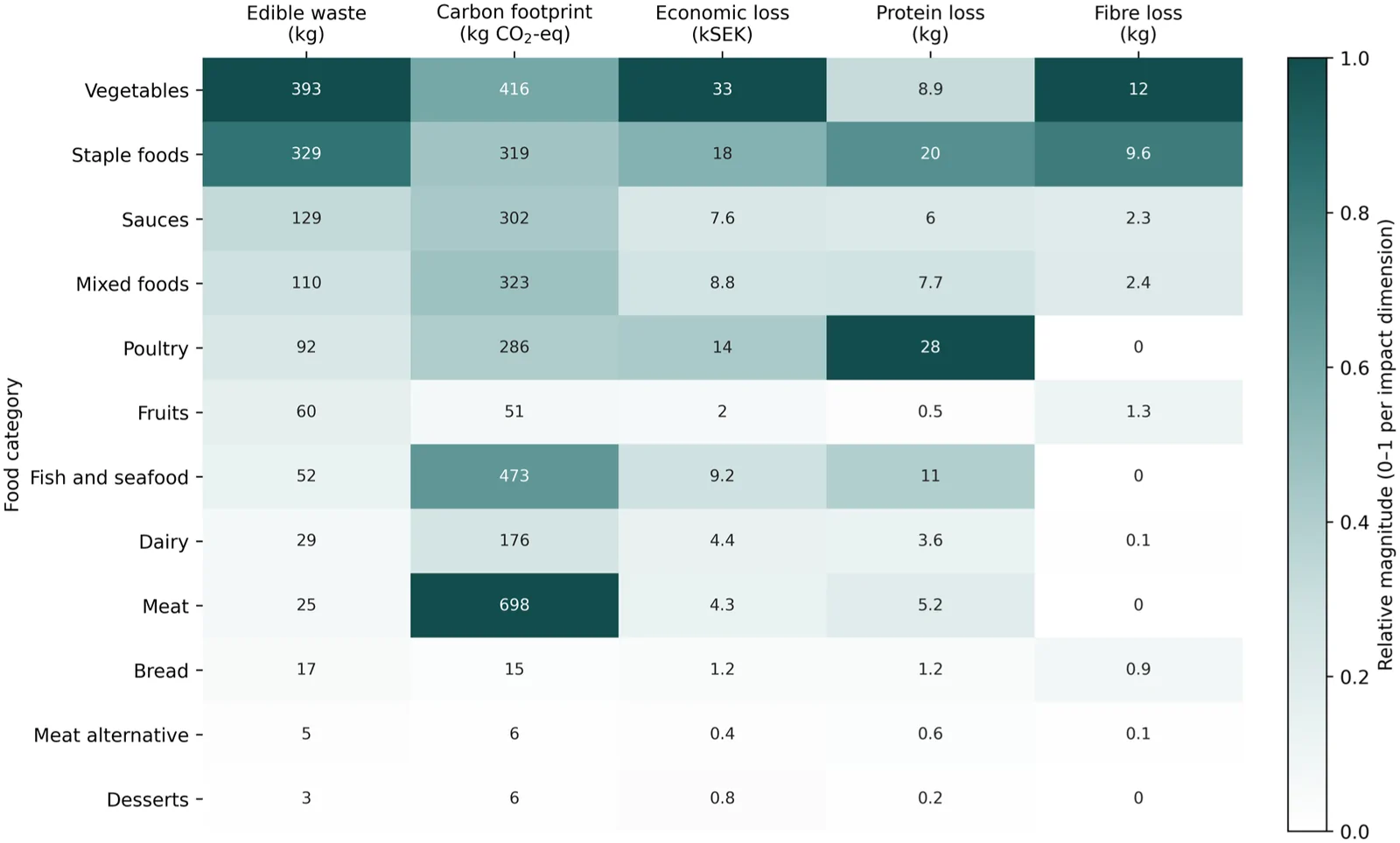
Edible food waste and associated sustainability impacts by food category. Heatmap showing the relative magnitude (0–1 per impact dimension) and absolute values of edible food waste and related sustainability impacts across food categories in the airline catering kitchen. Rows are ordered by total edible waste (descending). Darker shades indicate a higher relative contribution within each impact dimension. Values are normalised within each impact dimension (column-wise, 0–1).

Edible food waste varied substantially across food categories (Figure 1). Vegetables represented the largest fraction by weight (393 kg, 32% of edible waste), followed by staple foods (329 kg, 26%). In contrast, meat (24 kg) and fish and seafood (52 kg) contributed relatively little by mass but were disproportionately important in terms of sustainability impacts. When expressed as carbon footprint, meat, fish and seafood, poultry, and dairy together accounted for 53% of the total climate impact, despite representing only 16% of edible waste by weight. Vegetables and staple foods also contributed substantially to the overall footprint (~420 and 320 kg CO_2_-eq, respectively), reflecting their large volumes (Figure 1).

Also driven by their large volumes, the economic losses were highest for vegetables (33 kSEK (3000 EUR), 32%) and staple foods (18 kSEK (1620 EUR), 16%; Figure 1). Nutritional losses revealed distinct hotspots: protein losses were concentrated in poultry (28 kg, 30% of total) and staple foods (20 kg, 22%), while fibre losses were mainly associated with vegetables (12 kg, 42%) and staple foods (10 kg, 33%). The heatmap highlights a divergence between environmental and financial hotspots (Figure 1). While animal-based foods dominated the climate burden and protein losses, vegetables and staple foods emerged as the most consequential categories from an operational perspective, driving the majority of economic losses as well as fibre waste due to their high absolute volumes. This cross-dimensional comparison shows that no single food group dominated across all impacts.

### Qualitative insights

Staff interviews provided contextual explanations for the quantitative waste hotspots and revealed organisational dynamics shaping food waste in the airline catering kitchen. Three overarching themes emerged: (1) Food safety versus sustainability tensions; (2) Operational complexities and systemic gaps in coordination and information flow; and (3) Human factors in attitudes and practices.

#### Food safety versus sustainability tensions

Participants consistently emphasised the dominance of food safety rules in dictating discard decisions. Strict shelf-life regulations, plating limits, and thawing restrictions were described as non-negotiable, even when food appeared edible:

“You need to consume in 24 hours after thawing.” (JD I)

While staff recognised these measures as essential for passengers’ food safety, they also viewed them as a structural driver of trolley waste and short shelf-life losses. Several noted that company rules often exceeded aviation requirements, reinforcing an institutional culture of caution:

“. . . it’s something that has been defined by the headquarters in [another country], and that is applied in all the units.” (JD III)

“If the aviation is here, so [units] have a little bit above the standards for itself, sauce has something, but company has something over, so that’s why it’s a bit more.” (JD IV)

These accounts illustrate the tension between compliance with food safety standards and the aspiration to reduce waste, showing how overly conservative corporate policies can limit flexibility even when food remains fit for consumption.

#### Operational complexities and systemic gaps in coordination and information flow

Staff described a range of interrelated challenges that constrained efficiency in daily kitchen work. These occurred at two interconnected levels: operational complexities, that is, day-to-day difficulties, such as ad-hoc coordination and time pressure, and systemic gaps, referring to built-in weaknesses in organisational structures and information flows that made inefficiencies in forecasting, coordination, and waste-prevention recurrent.

Staff reported mismatches between planned and actual yields, time pressure, and communication gaps between departments. For instance, one participant explained how inaccuracies in production data created imbalances:

“Child and plating suddenly have too much left or too short of some items, so you have a yield [gap] there. It’s not correct. There is some yield implemented in the system, but it’s not accurate 100% because it is being done rushed by a very short time by the head chefs and head design.” (JD I)

Forecasting mismatches were recognised as a recurring systemic problem:

“The more difference between forecast and reality, so we make more waste as well, especially these sensitive [perishable] items.” (JD IV)

Staff also referred to production buffers that led to unavoidable surpluses:

“Many times, it shows they pushed over to the thawing room . . . what happens with this over if they can’t consume, we can’t send it to flight, and they can’t eat it, for example 100 extra pieces? What should we do with this hundred? We eat OK 20 or 30. The rest will be wasted.” (JD I)

Forecasting uncertainty was further exacerbated by last-minute client or crew changes, which often forced the disposal of already prepared meals:

“. . .they need crew, they need 300 people. . . .but next day they change to 200 so we can do nothing with this. They cannot use [the food] for another cycle.” (JD VIII)

“The demand of the crew that it takes until almost last-minute hours to order. It’s making this unbalance. . .” (JD I)

Alongside internal planning and coordination issues, external factors such as last-minute client changes, short supplier shelf life, and variable product quality further contributed to waste.

“Not good quality. Yes. Yes, we do claim . . . send to them, and they reply to us, OK. Send to us or send back or throw away, and we can pay for it.” (JD II)

Communication barriers were another common issue undermining coordination:

“She [a colleague from another department] came to me for vegetables, and I told [asked] her how much quantity she needs. She said she doesn’t know. . . that it’s my problem to find out.” (JD VIII)

Together, these operational and system-level constraints explain the quantitative hotspots observed in hot kitchen, trolley waste, and crew meals, both shaped by forecasting uncertainty, limited cross-department coordination, and the need to maintain production buffers under strict safety and service constraints.

#### Human factors in attitudes and practices

Most employees expressed strong awareness of the problem and a personal aversion to wasting food:

“It’s horrible. It’s horrible. I get sad.” (JD I)

“It’s not a good feeling someone hungry can eat that. . .” (JD VI/VII)

Participants referred to stock-rotation routines such as FIFO (First-In–First-Out) and expiry-first rotation, although adherence was described as inconsistent in busy times:

“And we’ll talk every morning. We’ll talk about it all up before. Take care about FIFO, yes.” (JD II)

“. . .they [kitchen staff] don’t check FIFO. They need to do fast job and they go in the fridge and take first one. . .” (JD II)

Motivation and communication within teams were seen as uneven, with some staff highly engaged while others were less proactive.

“. . .maybe because of laziness, but then I will say because they have a lot that they are doing, and they think at the same time. So, it becomes time-consuming to . . . It happened before a lot, but now it’s happening less and less because we’re trying to have less [waste]” (JD V)

Nevertheless, initiatives such as repurposing surplus food for staff meals and laboratory testing to verify extended shelf life were regarded as positive steps towards reduction.

Together, the interviews showed that food waste is structurally embedded at the intersection of safety regulations, forecasting uncertainty, and organisational culture. These insights help to explain why the hot kitchen and trolley sections emerged as multi-dimensional hotspots, why crew and business meals were particularly affected by operational volatility, and why vegetables and staple foods were dominated by weight and cost. To illustrate this integration between the quantitative and qualitative strands, Appendix Table A1 summarises the main waste hotspots identified through the waste audit together with explanatory themes and verbatim staff quotations.

## Discussion

This study provides one of the first integrated analyses of food waste generation in an airline catering kitchen, triangulating waste audits and staff interviews to identify hotspots, drivers, and sustainability implications. Most of the recorded waste was edible (89%), corresponding to about 10% of the total food produced during the study period. The observed waste rate was lower than values reported in some institutional catering contexts, such as hospital food services, where substantially higher waste levels have been observed, but falls within the broader range reported across food service settings, although direct comparisons are complicated by differences in system boundaries and reporting metrics, and the limited availability of published benchmarks from airline catering kitchens (Dias-Ferreira et al., 2015; Eriksson et al., 2017b; Filimonau and De Coteau, 2019). Moreover, the waste was concentrated in specific stages and categories: the hot kitchen, trolley waste, and crew meals, revealing targeted opportunities for prevention in specific process stages. Yet, realising this potential depends on overcoming organisational and regulatory constraints that limit flexibility in production planning and food handling. These findings advance previous research on flight-catering waste (e.g., Blanca-Alcubilla et al., 2018; Thamagasorn and Pharino, 2019) by shifting attention upstream from cabin waste management to kitchen-stage losses, where preventive potential is greatest, because food remains under the caterer’s control before service, allowing corrective action through, for example, improved forecasting, storage, and portioning (Eriksson et al., 2017b; Papargyropoulou et al., 2014).

A large proportion of the food waste in airline catering was edible and occurred after production but before service, reflecting inefficiencies embedded in planning and operational systems. The dominance of hot kitchen and trolley waste illustrated how overly strict food safety routines, forecast buffers, and limited flexibility interact to produce losses at the end of the production chain. Because these meals had already been cooked, their discard reflected not only the embodied production impacts but also wasted labour and energy invested in cooking, amplifying both environmental and financial losses. Similar tensions between sustainability and precautionary food safety standards have been documented in institutional food service settings (Dias-Ferreira et al., 2015; Eriksson et al., 2017b). In airline catering, just-in-time production, security protocols, and client variability further magnify these constraints, reflecting the tightly coupled and time-sensitive nature of airline catering supply chains (Rajaratnam and Sunmola, 2021a, 2021b).

Among these constraints, unpredictable demand emerged as the main underlying factor driving food waste generation in aviation catering. A significant driver of waste in crew and business meals was last-minute changes by airline clients or crew, which required buffer volumes or led to discards once production was underway. Participants reported changes up to less than 24 hours before departure, limiting flexibility. This volatility aligns with findings that demand inflexibility and uncertainty are core constraints in airline catering (Thamagasorn and Pharino, 2019). From a lean perspective, these patterns represent the waste of overproduction, preparing more than confirmed demand, leading to excess inventory and obsolescence (Gładysz et al., 2020). Lean literature identifies overproduction as the most critical of the “seven wastes,” since it cascades into further storage, handling, and discard losses. Further, the persistence of last-minute changes reflects, within the scope of this case study, structural barriers: client expectations of flexibility, contractual clauses allowing adjustments to protect passenger satisfaction, and reputational risks associated with under-supply. Similar challenges have been reported in the catering and events sector, where waste often arises from contractual over-commitment and guest no-shows (Tomaszewska et al., 2021; WRAP, 2015).

Beyond demand fluctuations, the qualitative findings also highlighted forecasting mismatches, short supplier shelf lives, and poor coordination between supply and production as major upstream constraints. Similar inefficiencies have been reported in institutional and commercial foodservice operations, where inaccurate forecasting, weak communication between departments, and limited managerial accountability contribute to production-stage waste (Betz et al., 2015; Filimonau and De Coteau, 2019; Papargyropoulou et al., 2016). While these inefficiencies arise mainly within kitchen operations, similar patterns, in this specific operational context, extend along the food supply chain. Similar contractual dynamics have been described in the retail sector, where supplier take-back agreements can obscure responsibility for pre-store food waste (Eriksson, 2012; Eriksson et al., 2017a). Although airline catering operates under different supply conditions, the same underlying issue of responsibility allocation determines whether losses materialise upstream or downstream. In the present study, inputs could not be returned, meaning that perishability risks manifested as visible kitchen-stage waste. However, evidence from agri-food supply chains indicates that clearly defined accountability and shared waste-reduction targets can improve coordination between supply and demand and reduce overproduction and waste (Ciliberti et al., 2022; Mohamadi et al., 2025).

Interpreting the identified hotspots through the food waste hierarchy (European Commission, 2020) highlights differences in prevention potential. Waste generated upstream, in hot kitchen operations, is largely avoidable and directly controllable, indicating strong opportunities for prevention through improved forecasting and production planning. Trolley and crew-meal waste are also, in principle, preventable, but are more strongly shaped by operational uncertainty, safety requirements, and service constraints, which limit the extent to which prevention can be realised in practice. In addition, opportunities for redistribution for human consumption are limited due to food safety regulations and operational constraints, meaning that these waste streams are more likely to be directed towards recovery processes such as anaerobic digestion or energy recovery, which are common food waste treatment pathways in Sweden (Naturvårdsverket, 2023).

The integrated sustainability assessment revealed clear cross-dimensional trade-offs: animal-based foods (meat, fish, poultry) entailed the highest carbon and protein impacts, while vegetables dominated waste mass, cost, and fibre losses. This divergence highlights that the heaviest waste streams are not necessarily the most environmentally significant, a finding consistent with observations from public catering contexts (Sundin et al., 2024a). For a profit-oriented company under decarbonisation pressure, this misalignment between economic and environmental priorities presents a strategic dilemma. Reducing waste in different food categories would have uneven sustainability implications: targeting high-volume categories such as vegetables would yield substantial economic savings and reductions in fibre losses, but comparatively smaller climate benefits per unit, whereas reducing smaller quantities of animal-based foods would deliver disproportionate climate and protein savings despite limited effects on total waste mass or cost. In this context, staple foods emerged as a balanced hotspot, combining high edible mass with substantial economic, environmental, and nutritional implications, and therefore representing a potential leverage point where reductions could deliver benefits across multiple sustainability dimensions simultaneously. These differences further imply potential co-benefits and unintended consequences: measures targeting high-volume waste streams may simultaneously reduce costs and improve resource efficiency but risk overlooking high-impact emissions, whereas focusing on animal-based waste may maximise climate benefits but offer limited financial incentives, potentially affecting implementation feasibility. As such, prioritisation strategies depend on whether the objective is cost efficiency, climate mitigation, or nutritional outcomes, underscoring the need for multi-criteria approaches. This is particularly relevant for EU food waste monitoring and SDG 12.3, which rely largely on aggregated food service data and may obscure such cross-dimensional differences.

This study was among the first to quantify nutrient losses associated with kitchen-stage waste in airline catering, providing a broader understanding of the social implications of food waste in this context. Beyond its environmental and financial impacts, the discarded edible food waste represented a complete loss of nourishment and public-health potential (Spiker et al., 2017). Per kilogram of edible waste, the estimated losses of protein (70 g) and dietary fibre (20 g) exceeded those reported for plate waste in school catering (57 g; 19 g, respectively; Sundin et al., 2024a), highlighting the high nutritional value embodied in kitchen-stage food waste. Unlike plate waste, which represents only the uneaten portion of served food, kitchen-stage waste reflects the full discard of food that never fulfilled its nutritional or service function. The finding that staple foods contributed notably to protein losses, despite their moderate protein density, demonstrated that nutritional inefficiencies can arise from large-scale waste of even low-climate-impact foods. Moreover, per kilogram of edible waste, the estimated climate impact (2.5 kg CO_2_-eq kg^−1^) was comparable to the catering-sector average (3.4 kg CO_2_-eq kg^−1^; Meier et al., 2021) but substantially higher than for school meal plate waste (1.0 kg CO_2_-eq kg^−1^; Sundin et al., 2024a). Together, these results indicate that kitchen-stage waste not only embodies substantial environmental impacts but also nutrient losses, highlighting the importance of upstream waste prevention and the inclusion of health-oriented objectives in future sustainability evaluations (Wang et al., 2023).

Translating these findings into practice requires coordinated action at both operational and systemic levels. Although stock-rotation practices, such as FIFO, were known, adherence was inconsistent under time pressure, a pattern common in high-paced catering operations. Operationally, inventory management based on FEFO principles can better align product use with remaining shelf life, provided there is robust information sharing across departments (Herron et al., 2022). Integrating predictive analytics and AI-based monitoring systems for demand forecasting and real-time waste tracking offers further opportunities to minimise perishable losses (Birkmaier et al., 2024; Sikander et al., 2026). Improved ICT connectivity and coordinated inventory systems have also been shown to reduce waste in perishable and catering operations (Nikolicic et al., 2021; Wu et al., 2021). Introducing more flexible menu design, where meal elements can be flexibly combined or repurposed, may further increase adaptability under uncertainty (Lévesque et al., 2022). However, digital and operational improvements alone may be insufficient if systemic issues are ignored and underlying contractual structures continue to permit volatile ordering practices.

From the systems perspective, a potential mitigation lies in revising contractual arrangements between catering providers and airline clients. Service-level agreements could, in principle, set clearer cut-off times for order modifications and limit the scope of last-minute changes, thereby aligning production volumes with confirmed demand. Evidence from event and contract catering indicates that inflexible contractual obligations can themselves contribute to overproduction and waste (WRAP, 2015). While overproduction in catering has also been linked to large or late orders and poor forecasting (Tomaszewska et al., 2021), preventive clauses are rarely enforced, as flexibility is often prioritised to maintain client satisfaction and reputational reliability. Comparable power asymmetries and service quality pressures have been documented across contract and institutional catering, where rigid agreements restrict suppliers’ ability to impose waste-reducing measures (Filimonau and De Coteau, 2019; Tomaszewska et al., 2021). In airline catering, where service quality and punctuality are critical, such contractual reform faces comparable barriers, suggesting that systemic change may require both client engagement and regulatory incentives to make waste-reduction commitments commercially viable. However, implementing such measures may entail upfront investments related to forecasting systems, process adjustments, and staff training, even if these may be offset over time through reduced waste and improved efficiency. Moreover, catering providers may face resistance from airline clients and competitive pressures to maintain flexibility, which can limit the feasibility of stricter waste-prevention practices.

While these practical measures provide potential pathways for action, several limitations should be acknowledged. The analysis was based on a single case study in Sweden, limiting the generalisability to other aviation catering facilities with different operational practices or regulatory conditions. The waste audit covered 21 weekdays in March 2025 during the low travel season, which does not account for seasonal variations. This implies that the identified hotspots and operational drivers should be interpreted as context-specific, and their magnitude may differ under other operational conditions, particularly during peak travel periods with higher production volumes. Future studies across multiple sites and seasons are needed to assess the robustness and transferability of the findings.

Moreover, retrieving food from mixed waste bins may introduce uncertainty due to potential contamination, misclassification, or incomplete recovery of discarded items. The presence of an “unknown” waste category further introduces some uncertainty in the allocation of waste across operational stages; however, given its relatively small share, its influence on the overall results is likely limited.

The quantification included only in-house prepared foods, excluding uplift and bank waste handled by the transport department, as well as frozen and ready-to-eat meals sourced externally for short-haul and international flights. These categories may represent additional waste volumes but were outside the economic and operational responsibility of the case kitchen. The presence of the researcher during waste audits may have influenced staff behaviour (Hawthorne effect), potentially leading to temporarily reduced waste levels compared to routine operations. The hotspot analysis was based on absolute waste quantities and did not account for differences in production volumes across kitchen sections, which may influence the interpretation of operational efficiency and relative performance between sections. Future studies should normalise waste generation by production output (e.g., per meal or per kilogram produced) to enable more precise comparison of operational efficiency across sections.

The sustainability assessment focused on carbon footprint, procurement cost, and two nutritional indicators; other environmental and social aspects, such as land and water use, micronutrient losses, or labour dynamics were not included. The environmental assessment relied on consumption-weighted average emission factors for foods on the Swedish market, and therefore did not explicitly account for variations in sourcing origin (e.g., local vs imported ingredients), which may introduce some uncertainty in the estimated impacts. A further limitation concerns the economic assessment, as procurement price data could not be disclosed due to commercial confidentiality constraints, which limits the independent verification of the economic estimates, particularly at the food category level. The qualitative findings, though rich in operational insight, reflect a small number of interviews and should be interpreted as exploratory. Nevertheless, the mixed-methods approach provided a robust and contextualised picture of kitchen-stage waste generation and its drivers. Finally, potential rebound effects could also be considered; however, existing evidence is largely limited to consumer contexts, and their relevance at the operational level of airline catering remains uncertain, warranting further research (Hegwood et al., 2023; Sundin et al., 2022).

Beyond these operational and contractual measures, the qualitative insights also revealed a cultural dimension of food waste. Staff expressed dislike over wasted food and a sense of satisfaction when waste was avoided, highlighting its emotional significance in daily work. Similar observations have been made in other hospitality settings, where staff attitudes, competence, and social norms strongly influence waste-prevention behaviour (Filimonau et al., 2024). Collectively, these studies show that food waste prevention is not only a technical or managerial challenge but is also shaped by organisational culture, shared norms, and motivation (Chawla et al., 2022). In this case, the strong emotional engagement expressed by staff suggests that motivation to reduce waste already exists but is constrained by procedural and organisational barriers. The challenge is therefore less about awareness and more about enabling agency by creating systems that allow employees to act on their environmental values within safety and operational limits (Lu and Ko, 2023). Enabling such agency requires clear corporate sustainability strategies and managerial support that empower staff to identify and act on waste-reduction opportunities while maintaining compliance with food safety standards (Chawla et al., 2022; Filimonau and De Coteau, 2019).

## Conclusions

This study provides one of the first integrated analyses of upstream, that is, kitchen-stage, food waste in airline catering, addressing an area that has so far received little attention compared with downstream cabin waste. While based on a single case study, the findings provide context-specific insights into upstream waste generation and its drivers in airline catering operations. Targeting upstream waste proved particularly important, as it was mostly edible (59 kg/day; 89% of total waste; 9% of total food produced) and occurred before meals reach passengers, representing more easily avoidable waste of fully prepared, resource-intensive food (2.5 kg CO_2_-eq, €7.4, 70 g protein, and 20 g fibre per kilogram waste). Unlike cabin waste, arising from passenger behaviour beyond the caterer’s control, kitchen-stage waste was mainly driven by overly strict food safety routines, late order changes, forecasting errors, and communication gaps. Revealed by the sustainability assessment, vegetable waste drove most economic and fibre losses, whereas animal-based waste entailed the highest climate impact and protein losses, indicating trade-offs. Across all dimensions, however, vegetables and staples emerged as recurrent hotspots, calling for balanced, system-wide prevention. Revising service-level agreements to include clearer cut-off times for order changes and shared waste-reduction targets integrated with food safety management and forecasting systems may help reduce systemic overproduction while preserving service reliability. Establishing policy frameworks and procurement guidelines that incentivise waste prevention and clarify shared responsibility among airlines, caterers, and suppliers will be essential to enable this transition.

## Supplemental Material

sj-pdf-3-wmr-10.1177_0734242X261455573 – Supplemental material for Food waste composition, hotspots and sustainability impacts in an airline catering kitchen Supplemental material, sj-pdf-3-wmr-10.1177_0734242X261455573 for Food waste composition, hotspots and sustainability impacts in an airline catering kitchen by Niina Sundin, Tahir Babatunde, Viachaslau Filimonau, Mattias Eriksson and Christopher Malefors in Waste Management & Research

sj-xlsx-1-wmr-10.1177_0734242X261455573 – Supplemental material for Food waste composition, hotspots and sustainability impacts in an airline catering kitchen Supplemental material, sj-xlsx-1-wmr-10.1177_0734242X261455573 for Food waste composition, hotspots and sustainability impacts in an airline catering kitchen by Niina Sundin, Tahir Babatunde, Viachaslau Filimonau, Mattias Eriksson and Christopher Malefors in Waste Management & Research

sj-xlsx-2-wmr-10.1177_0734242X261455573 – Supplemental material for Food waste composition, hotspots and sustainability impacts in an airline catering kitchen Supplemental material, sj-xlsx-2-wmr-10.1177_0734242X261455573 for Food waste composition, hotspots and sustainability impacts in an airline catering kitchen by Niina Sundin, Tahir Babatunde, Viachaslau Filimonau, Mattias Eriksson and Christopher Malefors in Waste Management & Research

## Appendix

**Table A1.** Integration of quantitative hotspots with qualitative explanations. The table links the main waste hotspots identified in the quantitative analysis with explanatory themes from staff interviews. Illustrative quotations (coded JD I–VIII) highlight how staff perceived the drivers of waste.

Quantitative hotspot	Interview explanation	Illustrative quotes
Hot kitchen and trolley waste	Strict safety rules, need for buffers, and forecasting mismatches created unavoidable surpluses.	“If the aviation is here, so newest [new units] have a little bit above the standards for itself, sauce has something, but new have something over, so that’s why it’s a bit more.” (JD IV) “The more difference between forecast and reality, so we make more waste as well, especially these sensitive [perishable] items.” (JD IV)
Crew and business meals	Last-minute airline or crew changes created demand uncertainty, leading to unavoidable surpluses.	“. . .they need crew, they need 300 people. . . .but next day they change to 200 so we can do nothing with this. They cannot use [the food] for another cycle.” (JD VIII) “The demand of the crew that it takes until almost last-minute hours to order. It’s making this unbalance. . .” (JD I)
Vegetables and staples (volume and cost)	Large volumes of vegetables and staple foods, combined with short supplier shelf lives and variable quality, led to economic and nutrient losses.	“Not good quality. Yes. Yes, we do claim . . . send to them and they reply to us, OK. Send to us or send back or throw away and we can pay for it.” (JD II) “No, but if I think directly that which thing is most, vegetable or something like that, it’s salads.” (JD VI/VII)
