# Supplementary material for: Food waste composition, hotspots and sustainability impacts in an airline catering kitchen
Source: Waste Manag Res. 2026 Jun 15;44(10):1703–16. doi: 10.1177/0734242X261455573 (PMC13612878; doi:10.1177/0734242X261455573)
Supplement: Supplementary file 1 [file sj-pdf-3-wmr-10.1177_0734242X261455573.pdf]

Semi-structured Interview Guide – Airline production kitchen

General Questions for All Staff

1. Perceptions of Food Waste

- What are your thoughts when you see food being wasted in the kitchen?
    - e.g. how do you feel about food being wasted?
- Do you think food waste is a significant issue in the kitchen (and other production sections)?
    - Why or why not?

2. Understanding the Waste Process

- What types of foods are most commonly wasted?
- At which stages of production do you think the most food waste occurs?
    - Why is that?
- Are there specific rules or guidelines for deciding when food should be discarded?
    - If so, who makes these decisions?

3. Current Practices

- Has anything been done to reduce food waste in the kitchen or in your department?
    - What was done, and did it help?
- Are there currently any practices that help reduce food waste in the kitchen or your department?
- Are there any actions or habits that you think contribute to increased food waste?
    - For example, are leftovers reused creatively in other menus?

4. Challenges and Barriers

- What do you think makes it difficult to reduce food waste in this kitchen or your department?
- Are there any particular challenges?
    - e.g. time, resources, or communication?

5. Motivations and Benefits

- What do you think would be the benefits of reducing food waste for the kitchen or the company?
  - How might reducing food waste affect staff workload, costs, or environmental impact?

6. Ideas for Improvement

- What ideas do you have for reducing food waste in this kitchen or your department?
- Are there any systems, tools, or policies that you think could help?
- Are there any systems, tools, or policies that contribute to food waste?
  - If so, why?
- Is there anything you would like to add?

Additional Questions for Management Staff

1. Existing Waste Management Systems

- Has this kitchen or department conducted a waste audit?
  - If so, what were the results?
- Is information currently collected on food waste (e.g. weighing bins or tracking waste types)?
  - How is this information recorded and used?

2. External Influences

- Are there any departments that influence how much food is wasted in your department?
  - Why/how?
- Is there anything you would like to add?
